# Comparison of the Effects of Different Food-Grade Emulsifiers on the Properties and Stability of a Casein-Maltodextrin-Soybean Oil Compound Emulsion

**DOI:** 10.3390/molecules25030458

**Published:** 2020-01-22

**Authors:** Yuan Liu, Zhen-Cheng Wei, Yuan-Yuan Deng, Hao Dong, Yan Zhang, Xiao-Jun Tang, Ping Li, Guang Liu, Ming-Wei Zhang

**Affiliations:** 1 Sericultural & Agri-Food Research Institute Guangdong Academy of Agricultural Sciences/Key Laboratory of Functional Foods, Ministry of Agriculture and Rural Affairs/Guangdong Key Laboratory of Agricultural Products Processing, Guangzhou 510610, China; liuyyuanx@163.com (Y.L.); zhencheng_wei@163.com (Z.-C.W.); yuanyuan_deng@yeah.net (Y.-Y.D.); zhang__yan_@126.com (Y.Z.); xjtang66@163.com (X.-J.T.); liping_apple@163.com (P.L.); 2 College of Food Science, South China Agricultural University, Guangzhou 510642, China; 3 College of Light Industry and Food Sciences, Zhongkai University of Agriculture and Engineering, Guangzhou 510225, China; dong.h@alu.scut.edu.cn

**Keywords:** food-grade emulsifiers, compound emulsion, succinylated monoglyceride, emulsion properties, emulsion stability

## Abstract

The improvement of food-grade emulsifiers in the properties and stability of complex emulsion has attracted much interest. In this study, the effects of six food-grade emulsifiers with a hydrophilic–lipophilic balance (HLB) range of 3.4–8.0 on a casein-maltodextrin-soybean oil compound emulsion were investigated by centrifugal precipitation rate (CPR), emulsifying activity index (EAI), microrheological properties, zeta potential, average particle size, and Turbiscan stability index (TSI). The optimal amounts of added succinylated monoglyceride (SMG) and polyglycerol fatty acid ester were 0.0025% and 0.1% (*w/w*), respectively, while that of the other four emulsifiers was 0.2% (*w/w*), according to the CPR. Thereinto, the SMG-stabilized emulsion exhibited the highest emulsifying activity and the lowest viscosity value and possessed the highest stability over 14 days of storage, which was indicated by the lowest TSI value and the smallest change in delta backscattering signal, relative to those of the other groups. Moreover, the emulsion stabilized by SMG displayed better emulsion stability than the control under a range of pH (6.0–8.0) and calcium ion concentrations (0–10 mM), which was attributed to the increased zeta potential value and the decreased average particle size of droplets with the addition of SMG. The present study provides a basic understanding for SMG improving the properties and stability of the complex emulsion.

## 1. Introduction

Oil-in-water (O/W) emulsions, which are a mixture of fine oil globules dispersed in the aqueous phase, are the basis of many food products such as milk, beverages, infant formulas, and foods for special medical purposes (FSMP) [1]. Although emulsions enable the food system to deliver nutrients, taste, and flavor, these applications are usually limited by their thermodynamics, which tend to break down during storage [2]. Gravitational separation (creaming, sedimentation) and droplet aggregation (flocculation, coalescence) are the most common emulsion destabilization phenomena [3]. These unstable phenomena will eventually lead to separation of the emulsion phase, thereby affecting the shelf life of the food emulsion.

One of the most effective ways of retaining emulsion stabilization is the addition of an emulsifier. Emulsifiers, containing both hydrophilic and hydrophobic functional groups, can alter the surface tension of a fluid to improve the dispersion of two incompatible solvents [4]. Other than the facilitation of emulsification, adding an emulsifier can also promote physical stability via adsorption at the oil–water interface, reducing the interfacial tension and preventing the droplets from aggregation [5]. Numerous types of emulsifiers including proteins, polysaccharides, phospholipids, and low-molecular-weight (LMW) emulsifiers can be used in the food industry [6]. Protein, one of the main nutrients in food, is also a natural emulsifier that stabilizes emulsions by forming a viscoelastic, adsorbed layer on oil droplets, thus developing a physical barrier to coalescence [7]. However, LMW emulsifiers do not form a viscoelastic surface. They exert an important effect on the composition of the interfacial layer. Adding a water-soluble surfactant after forming an interface or adding an oil-soluble surfactant before forming an interface will replace proteins at the interface and bind to proteins with various forces [8]. The types of small molecule emulsifiers such as nonionic, ionic, and amphoteric emulsifiers, all had an effect on the stability of the emulsion. Studies have shown that nonionic emulsifiers have better stability effects than ionic and amphoteric emulsifiers [9,10].

Compound emulsion systems such as milk beverages or FSMP consist of various nutrient components including proteins, carbohydrates, oils, minerals, and vitamins, and are sterilized using autoclaving, which increases the shelf life of compound emulsion products. Proteins in emulsion could facilitate emulsion stability, but thermal treatment cause protein denaturation, thus leading to emulsion destabilization [11]. The addition of LMW emulsifiers has become a suitable choice for improving the stability of a compound emulsion system [12,13]. The interaction between proteins and LMW emulsifiers in the formation and stability of emulsions has been previously studied. Jiang et al. reported that the interaction between LMW emulsifiers with caseinate decreased the particle size in the emulsion and improved the viscosity of the emulsion, and the nonionic emulsifier sucrose ester exhibited a stronger caseinate displacement and oil droplet absorption capacities than ionic and amphoteric emulsifiers [9]. Zhang et al. found that the combination of gelatin and surfactants such as lecithin or sodium dodecyl sulfate (SDS) was impacted by steric hindrance and hydrophilic–lipophilic balance (HLB) value [13]. Although these studies exist, fewer have focused on the effect of LWM emulsifiers on a compound emulsion (like FSMP-based formula emulsion) with autoclave sterilization treatment. Actually, this sterilized compound emulsion is closer to the actual emulsion in processing and production, and its stability is of more concern.

Therefore, the present study compared six food-grade emulsifiers with HLB values between 3.4 and 8.0 for their roles in the formation and stabilization of a caseinate-based compound emulsion (casein-maltodextrin-soybean oil). Considering the HLB value of sodium caseinate (HLB = 14) [14], the total HLB values of a protein-emulsifier mixture were in the range of 13.5–14.0, according to the calculation, which tended to form an oil-in-water (O/W) emulsion. Five LMW emulsifiers including four non-ionic emulsifiers and one amphoteric lecithin (LC), and one large molecular polysaccharide gum arabic (GA) were chosen to screen a suitable emulsifier for the preparation of the compound emulsion by the evaluation of the centrifugal precipitation rate (CPR), emulsifying activity index (EAI), microrheology, zeta potential, average particle size, and Turbiscan stability index (TSI), etc. Furthermore, the most suitable emulsifier against environmental factors including pH and ionic strength was also investigated. The present study aimed to provide theoretical guidance for the development and production of compound emulsions on the market.

## 2. Results and Discussion

### 2.1. Effects of Emulsifiers on the Centrifugal Precipitation Rate (CPR) of the Emulsion

**Figure 1:**
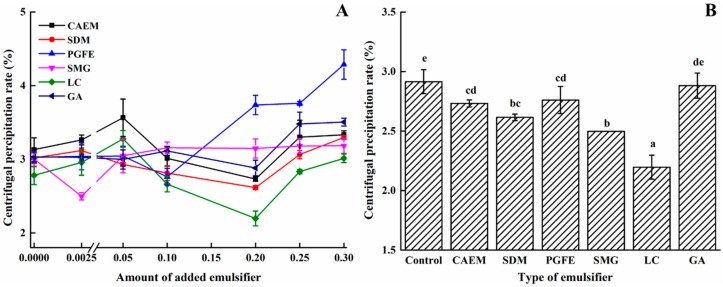
The centrifugal precipitation rate (CPR) of the emulsions with different emulsifier contents (**A**) and the CPR values of the six emulsifiers at their respective suitable addition levels (**B**).

In order to obtain the suitable amount of different emulsifiers added, the CPR of the emulsion was first investigated as it can reflect the physical stability of the emulsion to a certain extent. In general, the smaller the CPR value, the better the physical stability of the emulsion. Figure 1A shows the CPR changes for different amounts of added emulsifier. As shown in Figure 1A, the CPR values of all groups were between 2%–5%, and decreased first and then increased as the amount of emulsifier added increased, indicating that a suitable amount of each emulsifier could be acquired within the selected emulsifier concentrations (0–0.3%, *w/w*). The suitable addition level of polyglycerol fatty acid ester (PGFE) was 0.1% (*w/w*), while that of succinylated monoglyceride (SMG) was 0.0025% (*w/w*). Interestingly, the suitable addition amount of the other four emulsifiers was 0.2% (*w/w*).

Moreover, the CPR values of the emulsion corresponding to the suitable addition level of each emulsifier are shown in Figure 1B to facilitate the observation of the emulsifier effect. The addition of emulsifiers could significantly (*p* < 0.05) reduce the CPR values of the emulsions, except for the GA group. The LC emulsion exhibited the lowest CPR value (2.20%), followed by that of the SMG group (2.50%). The results indicate that LMW emulsifiers exhibited a better improvement in the physical stability of the emulsion than large molecular polysaccharides. The main reason might be that the LMW emulsifier possessed stronger oil droplet absorption capacity than large molecular polysaccharides, which reduced the particle size of the emulsion and thereby decreased the CPR values [15].

### 2.2. Effect of Different Emulsifiers on Emulsion Characteristics

The amount of added emulsifier obviously affected the oil–water interface film, which in turn impacted the emulsion stability. Therefore, the emulsions were prepared at the respective suitable amount of the emulsifier as described above. The characteristics including the emulsifying properties, microrheological properties, average particle size, microstructure, and TSI were further evaluated to choose the optimal emulsifier type in the selected emulsifiers.

#### 2.2.1. Emulsifying Properties

**Figure 2:**
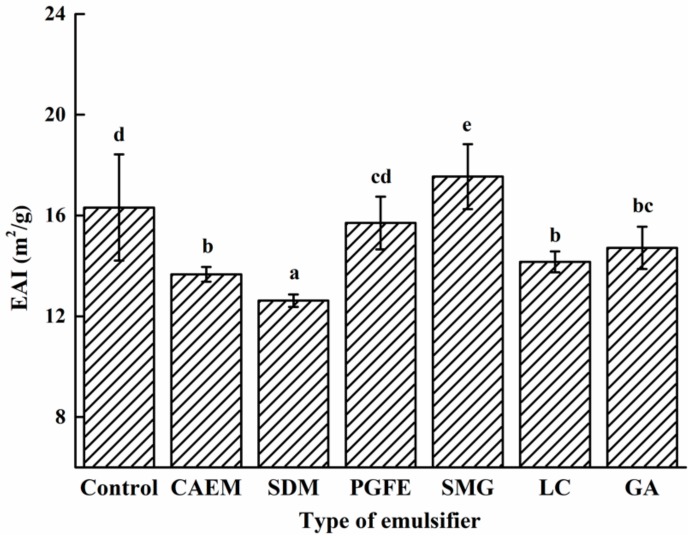
Effect of different emulsifiers on the emulsification activity index of the emulsions.

EAI is to express the emulsifying property of proteins in the oil/water interface area. In the present study, the effects of different emulsifiers on the emulsifying property of casein are shown in Figure 2. The EAI values of the different emulsion groups significantly (*P* < 0.05) changed after adding different emulsifiers. Except for the SMG group, the EAI values of the other five groups significantly decreased despite there being no statistical difference between the PGFE and control group. The emulsion with saturated distilled monoglyceride (SDM) had the lowest EAI value (12.62 m^2^/g). The reduced EAI values indicated that the supplementation of the emulsifiers negatively affected the absorption of casein to oil droplets, demonstrating a competitive adsorption between the emulsifier and casein. A similar result was reported by Susan et al., who found that both oil-soluble surfactant Span 60 and water-soluble surfactant Tween 60 could displace the protein molecules in the oil droplets interface [16].

**Figure 3:**
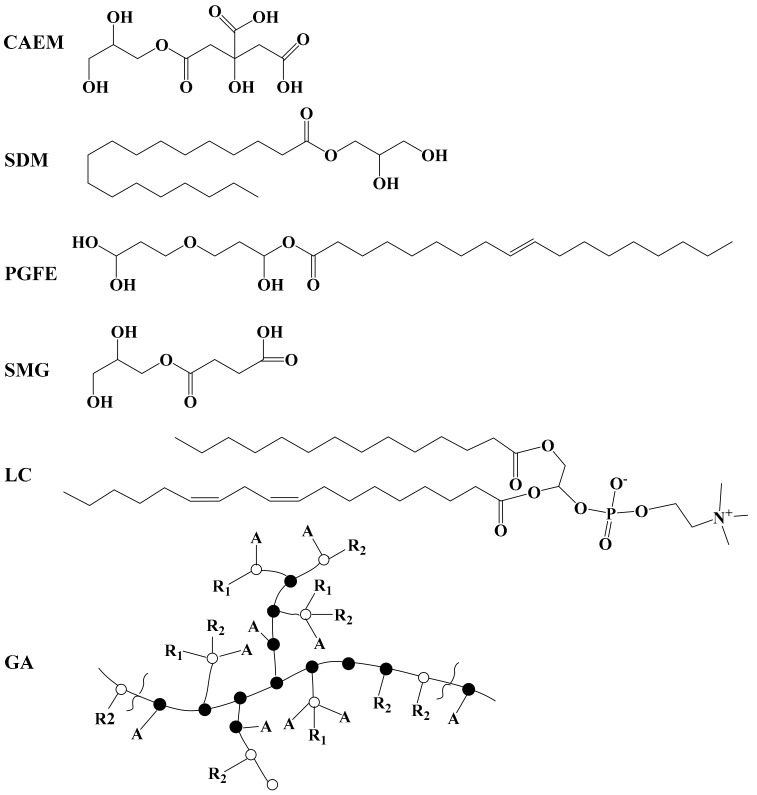
Molecular structures of citric acid ester of monoglyceride (CAEM), saturated distilled monoglyceride (SDM), polyglycerol fatty acid ester (PGFE), succinylated monoglyceride (SMG), lecithin (LC), gum arabic (GA). A = arabinosyl;●= 3-linked Gal*p* (Gal*p* attached);○= 6-linked Gal*p* (Gal*p* or Glc*p* attached), or end-group; R_1_ > Rha→ 4GlcA (Rhaoccasionally absent, or replaced by Me, or by Ara*f*); R_2_ = Gal→ 3 Ara; R_3_ = Ara→ 3 Ara→ 3 Ara...

However, of the six emulsifiers, the emulsion stabilized by SMG showed a higher EAI value (17.54 m^2^/g) than that of the control (16.31 m^2^/g), indicating that the presence of SMG increased the emulsifying activity of the casein, which implied that a synergistic effect was presented between SMG and casein instead of competition. The differences in the structure and composition of emulsifiers might lead to various EAI values [17]. Among the selected emulsifiers, SMG possessed the lowest molecular weight (192 Da) among the six emulsifiers, and had a lesser steric hindrance than the other emulsifiers (Figure 3), leading to the SMG absorbed in the oil droplet not displacing the protein (casein) in the oil droplet, showing a coordinated interface adsorption. Additionally, as an organic acid (–COOH) ester emulsifier, SMG could bind to protein, which facilitated the improvement of the emulsifying activity [18]. Although the citric acid ester of monoglyceride (CAEM) is another organic acid ester emulsifier, a longer lipophilic tail in CAEM prevented the casein and emulsifier from adsorbing to oil droplets at the same time, resulting in reduced emulsifying properties. Similar results were reported by Cheong et al. [19], who found that the emulsion produced by sodium caseinate and a smaller molecule emulsifier (Tween 20) had better properties than the emulsions stabilized by sodium caseinate. Mao et al. also found that small-molecule emulsifiers (decaglycerol monolaurate) were more capable of forming smaller droplets than large-molecule emulsifiers (octenyl succinate starch) [20].

#### 2.2.2. Microrheological Properties

**Figure 4:**
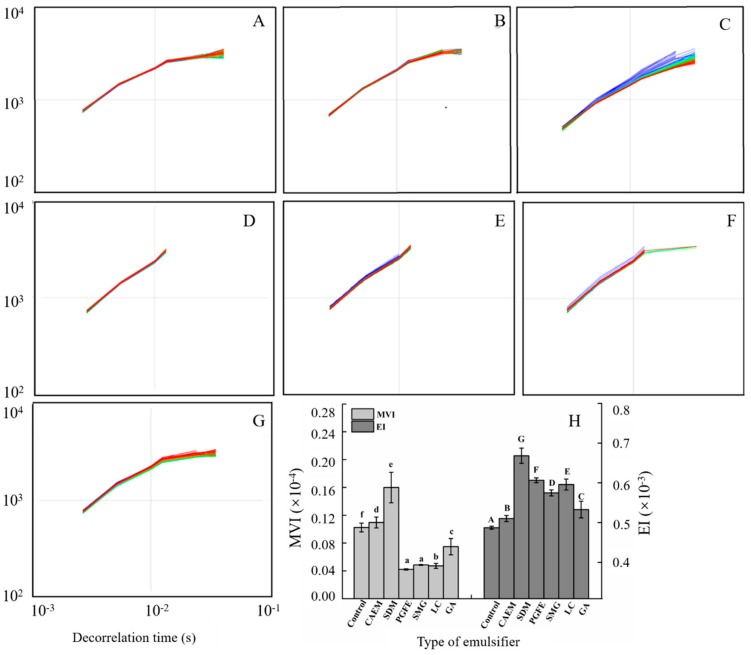
Effect of emulsifiers on the microrheological properties of emulsions. (**A**–**G**) mean square displacement (MSD) of the emulsions for the different added emulsifiers (control, CAEM, SDM, PGFE, SMG, LC, and GA); different colors and arrows refer to different MSD analysis scanning times. (**H**) elastic index (EI) and macroscopic viscosity index (MVI) values for emulsions with different emulsifiers. Different letters in the same indicator indicated significance (P < 0.05).

The microrheological technique, which does not alter the structure of the emulsion compared to classical mechanical rheology, was used to monitor the Brownian motion of droplets, tracking droplet interactions by microrheology [21]. The mean square displacement (MSD) curve reflects the trajectories of the system particles. The MSD of the droplets was a direct measure of the dynamic properties of the droplets and the emulsifier in which they were embedded. As shown in Figure 4A–G, all emulsions were purely viscous over the range of decorrelation time, giving an MSD that scaled linearly with the decorrelation time, which could be attributed to the free motion of granules. 

The elasticity strength and macroscopic viscosity of the samples were represented by the elastic index (EI) and macroscopic viscosity index (MVI) values obtained from the MSD curves, respectively. EI corresponds to the inverse of the distance traveled by droplets before interacting with the network, while MVI corresponds to the inverse of the particle speed over long periods of time [22]. Figure 4H shows the effect of different emulsifier types on the EI and MVI values of emulsions at 25 °C. The EI values of all groups with emulsifiers increased relative to that of the control, which indicated that the addition of emulsifiers improved the elasticity strength of the emulsions. However, the MVI values of emulsions stabilized by PGFE, SMG, LC, or GA significantly (*p* < 0.05) decreased, whereas those of the emulsions stabilized by CAEM or SDM increased. Although the PGFE group exhibited the lowest MVI values, no significant differences existed between the PGFE and SMG groups. A previous report showed that the interaction force in the system was tight and had a small viscosity, which was more suitable for tube feeding without tube plugging [23]. Therefore, tube feeding would ideally be applied to emulsions stabilized by PGFE or SMG. 

#### 2.2.3. Zeta Potential

**Figure 5:**
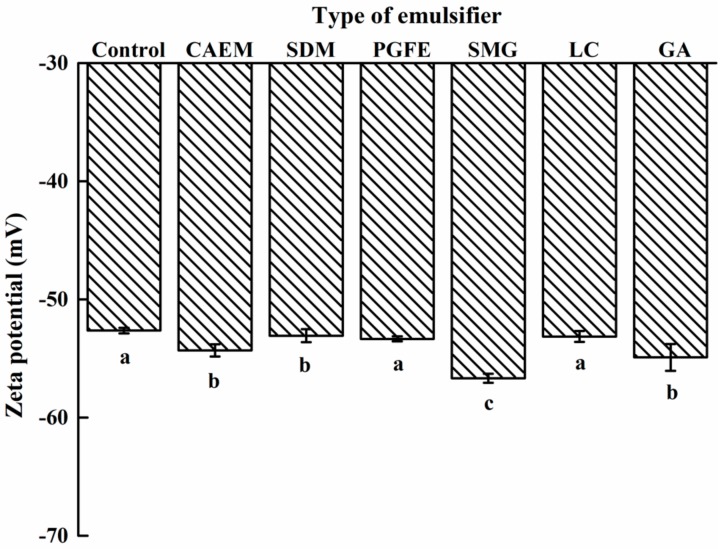
Effects of different emulsifiers on the zeta potential of emulsions.

The zeta potentials of the emulsions are shown in Figure 5. Since all emulsion groups were in the negative zeta potential region, the absolute value of the zeta potential was used for comparison. The absolute values of zeta potential of all additive groups was higher than that of the control, although no significance (*P* < 0.05) presented among the PGFE, LC, and control groups. The emulsion with SMG showed the highest absolute value of zeta potential (56.7 mV), which increased by 8% relative to that of the control (52.6 mV).

Generally, a suspension with an absolute zeta potential above 30 mV is considered to have good stability, and those above 60 mV indicate excellent stability and possible settling [24]. An increase in the zeta potential indicates a decrease in the attraction between droplets and an increase in the repulsive force. Hence, the emulsion stabilized by SMG exhibited the best stability among all groups. SMG is an organic acid monoglyceride emulsifier that might interact with sodium caseinate at the oil–water interface by the electrostatic action, which improves the zeta potential. 

#### 2.2.4. Average Particle Size

Stokes’ law states that the velocity at which a droplet moves is proportional to the square of its radius [25]. Therefore, the change in droplet size plays an important role in emulsion stability. Multiple light scattering, which could be utilized to determine the droplet size of an emulsion without dilution, was used to evaluate the effects of emulsifiers on the emulsion particle size.

**Figure 6:**
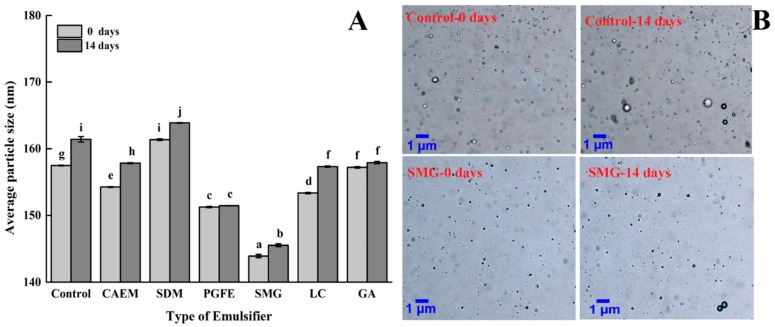
Changes in the particle size of different emulsifiers in the emulsion during storage. (**A**) The particle size of the different emulsifiers; (**B**) the optical micrographs of the control and succinylated monoglyceride (SMG)-stabilized emulsion on days 0 and 14.

As shown in Figure 6A, compared to the control, all additive groups showed smaller average emulsion particle sizes (<160 nm), except for the SDM group. The emulsion with SMG had the lowest average particle size (143.9 nm). After 14 days of storage, the average particle size of the PGFE and GA groups did not change significantly, while that of the other four groups obviously increased. Nevertheless, the average particle size of the emulsion stabilized by SMG still displayed the lowest value (145.5 nm), only a 1.1% increase after 14 days of storage. The results were further proven by the optical microscopy images (Figure 6B). In the selected emulsifiers, SMG had a HLB value of 6.0, which was lower than LC or GA (Table 1). However, the total HLB value of casein and the emulsifier mixture should be recalculated since the casein and emulsifier mixture were adsorbed on the surface of oil droplets at the same time. In this way, the HLB value of the SMG and casein mixture was recalculated as 13.994, which was the highest value among the six emulsifiers. According to a previous report, the higher the hydrophilic head-to-hydrophobic tail volume ratio, the higher the droplet curvature and the smaller the droplet size [26]. Therefore, the present results might be attributed to the synergistic effect of SMG and casein improving the droplet curvature, resulting in the smallest particle size of the emulsion compared to the other emulsions. Previous research has demonstrated that a smaller particle size indicated better emulsion stability [27]. Therefore, the results indicated that the SMG-stabilized emulsion had better stability, which was consistent with the result of the SMG-stabilized emulsion displaying the highest absolute zeta potential.

### 2.3. Evaluation of the Physical Stability of Different Emulsifiers in Emulsions by Turbiscan

To further evaluate the emulsion stability, Turbiscan analysis, which enables continuous monitoring of the optical properties of emulsions and hence provides real-time information on destabilization phenomena [28], was employed for this study. The indices of delta backscattered light value (ΔBS, ΔBS = BS_t_ − BS_0_) and TSI were recorded to estimate the physical stability of the emulsion system with or without different emulsifiers.

#### 2.3.1. ΔBS Curve of the Emulsion

**Figure 7:**
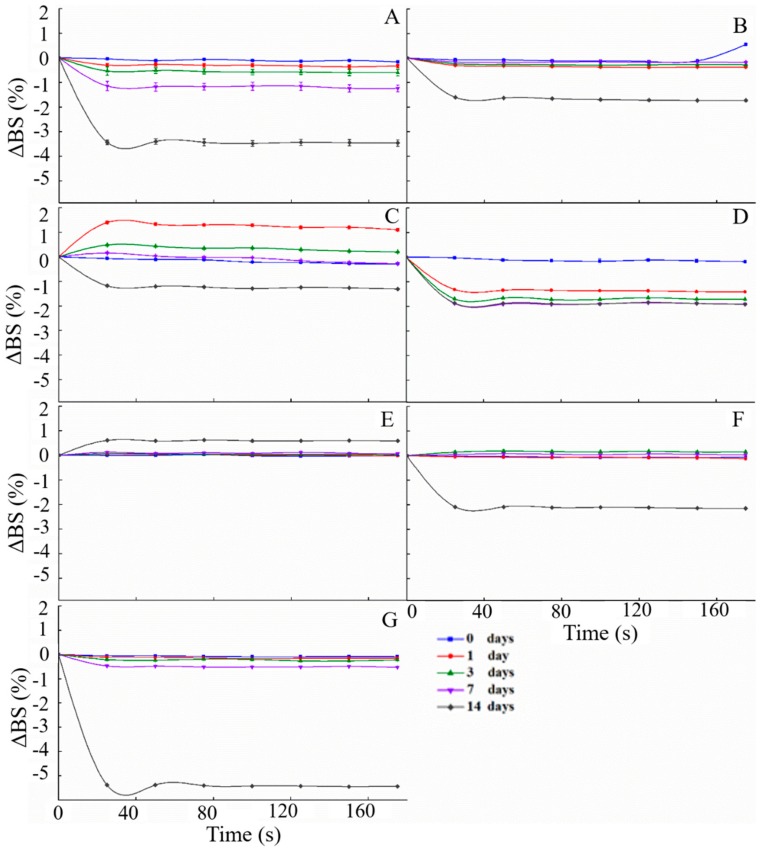
Changes in the backscattered light (BS) curves of different emulsifiers in the emulsion during storage. (**A**) Control group; (**B**–**G**) emulsions with added CAEM, SDM, PGFE, SMG, LC, and GA, respectively.

ΔBS is a parameter that is directly dependent on the droplet mean diameter and the droplet volume fraction. Thus, changes in the backscattering profile of the emulsion sample are related to the droplet size changes and migration processes. Usually, a positive ΔBS value indicates the migration of oil droplets, while a negative ΔBS value implies the accumulation of droplets [29]. Figure 7 shows the change in the ΔBS value of all emulsion samples during 14 days of storage. The ΔBS profile of the emulsion showed that the backscattered light value (BS) changed dramatically, demonstrating that the emulsion particles accumulated or floated during storage. With increased storage time, the BS of most samples gradually decreased, resulting in a negative ΔBS value (Figure 7A,B,D,F,G). This result indicates that the emulsion samples underwent phase separation, leading to a decrease in the oil droplet concentration, which in turn reduced the intensity of the backscattered light. Moreover, the ΔBS of the SDM-stabilized emulsion (Figure 7C) presented positive values during the seven days, but became negative on day 14, which revealed that the emulsion with SDM experienced the accumulation of droplets and then phase separation. The emulsion with SMG underwent the accumulation of droplets, indicated by the positive ΔBS values during storage. Among these groups, the SMG group exhibited the smallest range of changes in ΔBS values, increasing only by 0.6%, which further proved that the emulsion particles were evenly distributed and retained good stability [30].

#### 2.3.2. Emulsion Stability Index

**Figure 8:**
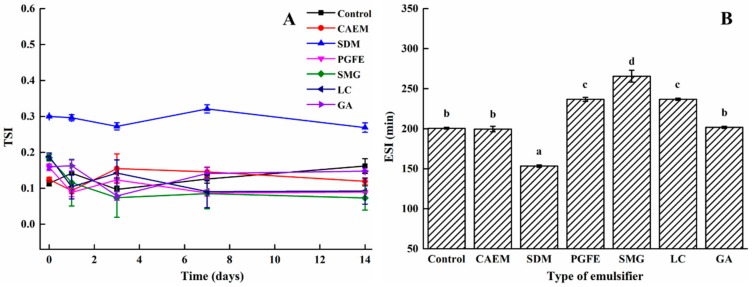
Turbiscan stability index (TSI) (**A**) and emulsifying stability index (ESI) (**B**) values of emulsions prepared with different emulsifiers.

TSI is a parameter that can be used to estimate the emulsion stability and is obtained as the sum of all destabilization phenomena including the thickness of the sediment and clear layer and the particle settling that occurs during the monitoring process [31]. The lower the TSI value, the better the system stability. The stability changes of the emulsion with/without different emulsifiers were recorded using the TSI value over 14 days of storage. Figure 8A shows that the TSI value of all samples was less than 0.5. The high value (> 0.5) of the TSI indicated the instability and high probability of phase separation, whereas a low TSI value indicated the stability and low probability of phase separation [32], so all emulsions obtained had good stability. Compared to the control group, all emulsions, except for the SDM group, yielded a lower TSI value over time. The SMG-stabilized emulsion showed the lowest TSI values during the entire storage period, indicating that SMG was the most effective emulsion stabilizer. Munk et al. [33] found that the presence of LMW emulsifier would affect the TSI value of the emulsion. This was because such emulsifiers could enter the surface of oil through small gaps, which hindered the aggregation between droplets and prevented phase separation. Compared with other emulsifiers, SMG had the smallest molecular weight and was easily adsorbed in the interstices of the oil–water interface. It also electrostatically bound to proteins, thickened the interface film, and enhanced the dispersion of the emulsion. Moreover, another stability parameter, the emulsifying stability index (ESI), was employed to further assess the stability of the emulsions. Unlike TSI, a larger ESI value indicates better emulsion stability. The ESI profiles of the emulsions are shown in Figure 8B. As expected, the SMG-stabilized emulsion had the highest ESI value among all groups, indicating the best emulsion stability, which was consistent with the TSI results. The ESI was measured by the turbidimetric method; therefore, the emulsion particle size significantly affected the ESI value. The highest ESI value (265.54 min) for the SMG-stabilized emulsion indicated the smallest emulsion particle size, which was in accordance with the results where the SMG-stabilized emulsion possessed the lowest average particle size (Figure 6).

In summary, the most suitable emulsifier for the casein-maltodextrin-soybean oil compound emulsion was SMG, according to the results related to the emulsion characterization and stability. Among the emulsifiers, SMG had the smallest molecular weight, which contributed to a synergistic emulsification effect with casein [34].

### 2.4. Effect of Calcium Ion Concentrations and pH on the SMG-Stabilized Emulsion

In a compound emulsion system, the ionic strength and pH are critical environmental factors that affect the stability of the emulsion beyond the emulsifier. Therefore, the present study further evaluated the influences of the calcium ion concentration and pH on the compound emulsion stabilized by SMG.

#### 2.4.1. Calcium Ion

A previous study reported that calcium ions significantly affected emulsion stability, with a low concentration of ionic calcium inducing flocculation of the casein-stabilized emulsion [35]. Therefore, this study also estimated the influence of calcium ions on the zeta potential and particle size of SMG-stabilized emulsions.

**Figure 9:**
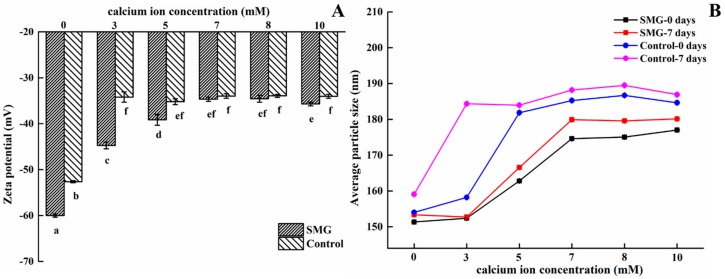
Effects of calcium ions on the stability of the SMG-stabilized emulsions. (**A**) Zeta potential of emulsions; (**B**) average particle size of emulsions.

Zeta potential measurements were first performed to evaluate the electrostatic deposition of calcium ions onto the interfacial film surrounding the droplets. Figure 9A shows that the zeta potential values of both groups obviously decreased with increasing calcium ion concentration (0–10 mM), implying a negative effect on emulsion stability via the addition of calcium ions, which was consistent with a previous report [36]. Calcium ions with a positive charge could adsorb to the surface of SMG-stabilized emulsion droplets with a negative charge by electrostatic interaction at the interface, resulting in a decrease in zeta potential. In the present study, the SMG-stabilized emulsion displayed higher zeta potential values at the same concentration of calcium ions than the control, suggesting a higher concentration of calcium ion tolerance.

A decrease in zeta potential implied a weakening of electrostatic interactions between the emulsion droplets, which in turn resulted in particle aggregation. As expected, the average particle size of both samples increased as the calcium ion concentration increased. The SMG-stabilized emulsion exhibited a lower particle size relative to that of the control sample, even after seven days of storage, which was attributed to the higher zeta potential of the emulsion with SMG (Figure 9B).

In short, the addition of calcium ions significantly changed the particle size and zeta potential of both emulsion samples. However, the absolute zeta potential values of both groups exceeded 30 mV within the selected calcium ion concentration range (< 10 mM), which indicated the good stability of the emulsions with or without SMG. Moreover, the SMG-stabilized emulsion exhibited better calcium ion tolerance than the control group.

#### 2.4.2. pH Values

**Figure 10:**
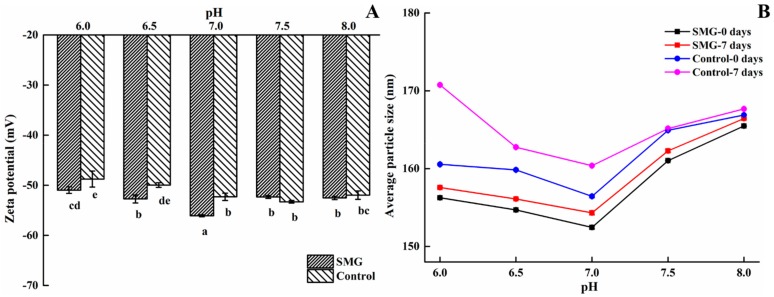
Effects of pH on the stability of SMG-stabilized emulsions. (**A**) Zeta potential of emulsions; (**B**) average particle size of emulsions.

The effects of pH on the zeta potential of the complex emulsion with/without SMG are shown in Figure 10A. Compared to the influence of calcium ions, pH only had a slight effect on the emulsion zeta potential. The zeta potential values of both samples first increased and then decreased with the increase of pH. When the pH was 7.0, both samples had the highest zeta potential values, −56.1 mV and −52.3 mV for the emulsion with and without SMG, respectively. 

Furthermore, the change in particle size of the SMG groups was determined at different pH values (6.0–8.0) over the course of seven days. In Figure 10B, a pH of 7.0 was the best to form the smallest particle size for both emulsions. The particle size of the emulsion stabilized by SMG remained smaller than that of the control sample, suggesting the better emulsion stability of SMG. Overall, neutral conditions (pH 7.0) were found to have a positive effect on the emulsion stability, which was consistent with the results by Owens et al. [37].

## 3. Materials and Methods

### 3.1. Materials

Soybean oil was purchased from a local market in Guangzhou. CN-EM7 casein sodium, made by drum drying, with 90% protein was purchased from Davidine International Trading Co. Ltd. (Shanghai, China). Dextrose Equivalent 15 (DE 15) maltodextrin was purchased from Shandong Xiwang Starch Co. Ltd. (Shandong, China). CAEM, SDM, PGFE, and SMG were purchased from Riken Vitamin Co. Ltd. (Shanghai, China). LC was purchased from Guangzhou Haisha Biotechnology Co. Ltd. (Guangzhou, China). GA was purchased from Taixing Dongsheng Food Technology Co. Ltd. (Taixing, China). All chemicals were of analytical reagent grade, and all solutions were prepared with deionized water.

### 3.2. Preparation of the Emulsion

4% (*w/v*) casein sodium (SC), 15% (*w/v*) DE 15 maltodextrin, and different emulsifiers (CAEM, SDM, PGFE, SMG, LC, and GA) were mixed and dissolved in distilled water with a high-speed homogenizer (IKA^®^ Works, Germany) at 10,000 r/min. Then, 3% (*v/v*) soybean oil was slowly added until complete emulsification was achieved. The pH of the emulsion was adjusted to 7.0 with sodium hydroxide. The mixed emulsion was then subjected to high-pressure homogenization (SHP-60 high pressure homogenizer, Shanghai Kesi Da homogeneous Electromechanical Equipment Co. Ltd., Shanghai, China) treatment and cycled twice under a pressure of 25 MPa to obtain the final emulsion. The final emulsion was dispensed into a glass bottle, sealed, and placed in a 121 °C autoclave for 20 min to obtain the emulsion required for the study, and the samples were stored at 25 °C for further analysis. The ranges of the addition amounts and the molecular weights of the six emulsifiers are shown in Table 1, respectively. Considering the HLB value of casein (HLB = 14), the total HLB (HLB_total_) of the emulsifier mixed with casein was calculated according to the mass fraction of the surfactant (X_i_) as follows:

(1) $$HLB_{\mathrm{total}}=\sum HLB_{i}•X_{i}$$

Subsequently, in the preparation of the emulsion, a suitable addition amount of six emulsifiers was separately added, and the emulsifier superior to the system was selected by exponential measurement.

### 3.3. Characterization of Emulsions

#### 3.3.1. Determination of the Centrifugal Precipitation Rate

The CPR was determined based on the method described by Jensen et al. [38] with slight modification. The samples (20 g, m_0_) were accurately weighed in a centrifuge tube (50 mL) and centrifuged at 6000 r/min for 15 min using a tabletop centrifuge (Thermo Fisher Scientific, Waltham, MA, USA). Afterward, the supernatant was discarded, and the precipitate (m_1_) was weighed. The CPR value was calculated using Equation (2).

(2) $$CPR\left(\%\right)=\frac{m_{1}}{m_{0}}\times 100$$

where m_1_ is the weight of precipitation after centrifugation and m_0_ is the weight of the sample (g), with a value of 20 g in this paper.

#### 3.3.2. Emulsifying Properties

The EAI and ESI of the emulsion system were determined by turbidimetry [39]. The freshly prepared emulsion was first diluted 100-fold with sodium dodecyl sulfate (0.1% SDS, *w/w*). The dilution absorbance was measured at 500 nm using an ultraviolet spectrophotometer (UV 1800, Shimadzu, Japan) with 0.1% SDS as a blank. The absorbance of the fresh emulsion (A_0_) and the emulsion after 10 min (A_10_) was then measured, and the EAI (m^2^/g) and ESI (min) were calculated using Equations (3) and (4), respectively.

(3) $$EAI\left(m^{2}/g\right)=\frac{2\times 2.303}{C\times \left(1-\phi \right)\times 10^{4}}\times A_{0}\times D$$

(4) $$ESI\left(\min\right)=\frac{A_{0}\times \Delta T}{A_{0}-A_{10}}$$

where D is the dilution factor; C is the protein concentration (g/mL) before protein emulsion formation (i.e., 0.04 g/mL); Φ is the volume fraction of the oil phase (L/L) (i.e., 0.03), and ΔT is 10 min.

#### 3.3.3. Microrheological Properties

The microrheological properties of emulsions with different emulsifiers added to them were measured by the Microrheolaser Lab (Rheolaser Master, Formulaction Inc., Toulouse, France) based on diffusing wave spectroscopy (DWS). Microrheological tests were carried out on different emulsifier-stabilized emulsion samples at 25 °C. The instrument used measures the Brownian motion of the particles as the particle MSD versus time. The EI and MVI of the samples were obtained by using RheoSoft Master 1.4.0.0.

#### 3.3.4. Microstructure Analysis

A light microscope (Olympus CX41, Tokyo, Japan) was used to characterize the morphology of the emulsions after the addition of different emulsifiers, and micrographs of the emulsions were taken at 100× magnification. At least three specimens of each sample were observed to obtain representative micrographs of the samples.

#### 3.3.5. Zeta Potential

The zeta potentials of the emulsion droplets were evaluated using a zeta potential analyzer (Zetasizer Nano-ZS 90, Malvern Instrument Co. Ltd., Worcestershire, UK). The samples were diluted 100-fold and then measured after equilibration at 25 °C for 120 s three times each.

#### 3.3.6. Determination of the Physical Stability of the Emulsion by Turbiscan

The emulsion stability was investigated using a Turbiscan instrument [40]. The emulsion (20 mL) was transferred to a cylindrical glass cell and scanned at 25 °C for 180 s. Furthermore, due to the high turbidity of the sample and the small amount of transmitted light, the BS was used in this experiment. The results are presented in terms of the ΔBS, the TSI, or the size of a dispersed droplet. 

### 3.4. Influencing Factors of Emulsion Stability

#### 3.4.1. Evaluation of Emulsion Stability Relative to Calcium Ions

The emulsions (pH 7.0) were formulated with different amounts of calcium ions (0, 3, 5, 7, 8, and 10 mM) according to the above method to form a series of emulsion samples. These samples were stored at 25 °C prior to analysis. The droplet diameter and zeta potential were measured, and then the samples were stored for one week prior to visual observation.

#### 3.4.2. Evaluation of Emulsion Stability Relative to the pH

The emulsions (pH 7.0) were dispensed into different beakers, and then the pH of each sample was adjusted to 6.0 to 8.0 at intervals of 0.5 using a HCl or NaOH solution. The samples were stored at 25 °C prior to analysis. The droplet diameter and zeta potential were measured, then the samples were stored for one week prior to visual observation.

### 3.5. Statistical Analysis

Data were processed using Origin 9.0 and SPSS 22.0. The Duncan method was used to compare the differences between groups. The significance level was *P* < 0.05. All experiments were repeated three times.

## 4. Conclusions

This study demonstrated the effect of protein and emulsifier recombination on stability in the emulsion formula. The CPR value indicated that as the concentration of the emulsifier increased, the stability of the emulsion decreased first, and then increased. All five small-molecule emulsifiers and large-molecule arabinose could interact with proteins at the oil droplet interface, affecting the emulsifying properties of casein. Only SMG showed a positive effect on the emulsifying properties. The addition of emulsifiers improved the elasticity strength of the emulsions, while only emulsions with PGFE or SMG decreased its viscosity, which promote their applications in FSMP to easy tube feeding. Among the selected emulsifiers, the emulsifier treated with SMG exhibited the smallest average particle size and the largest absolute value of the zeta potential, indicating the best stability of the emulsion. This speculation was further proved by the results from the light scattering assay, with the lowest TSI value and the smallest change in delta backscattering signal for the SMG emulsion relative to those of the other groups during 14 days of storage. Moreover, in the range of pH (6.0–8.0) and calcium ion concentration (0–10 mM), the SMG-stabilized emulsion showed better emulsion stability than the control group, and a pH of 7.0 was found to have the most positive effect on the emulsion stability. Overall, SMG was the most suitable emulsifier in the model system. Compared to other emulsifiers, SMG had the lowest molecular weight (192 Da) and least steric hindrance, which promoted its interaction with oil droplets without displacing protein. Additionally, the mixture of SMG and casein had the highest HLB value (13.994) relative to the other groups, which improved the droplet curvature and thereby decreased particle size, facilitating the stability of the emulsion. Nevertheless, we also expect that future works could focus on the detection of interfacial tension and surface protein concentration, which will be beneficial for understanding the structure, function, and application of SGM-stabilized emulsions. Further work is also necessary to explore the application of SMG-stabilized compound emulsions in different food products.

## Figures and Tables

**Table 1:** The selected amounts of emulsifier added.

Type of Emulsifier	Molecular Formula	Relative Molecular Mass (Da)	HLB	Total HLB Value	Addition Amount /%
Citric Acid Ester of Monoglyceride (CAEM)	C_9_H_14_O_9_	266	3.4	13.495	0, 0.0025, 0.05, 0.10, 0.20, 0.25, 0.30
Saturated Distilled Monoglyceride (SDM)	C_21_H_42_O_4_	358	4.3	13.538	
Polyglycerol Fatty Acid Ester (PGFE)	C_24_H_46_O_6_	430	5.5	13.793	
Succinylated Monoglyceride (SMG)	C_7_H_12_O_6_	192	6.0	13.995	
Lecithin (LC)	C_42_H_80_NO_8_P	758	7.0	13.667	
Gum Arabic (GA)	\	220,000	8.0	13.714	
